# Host blood RNA signatures predict the outcome of tuberculosis treatment

**DOI:** 10.1016/j.tube.2017.08.004

**Published:** 2017-12

**Authors:** Ethan G. Thompson, Ying Du, Stephanus T. Malherbe, Smitha Shankar, Jackie Braun, Joe Valvo, Katharina Ronacher, Gerard Tromp, David L. Tabb, David Alland, Shubhada Shenai, Laura E. Via, James Warwick, Alan Aderem, Thomas J. Scriba, Jill Winter, Gerhard Walzl, Daniel E. Zak, the Catalysis TB–Biomarker Consortium, Nelita Du Plessis, Andre G. Loxton, Novel N. Chegou, Myungsun Lee

**Affiliations:** l Department of Science and Technology, National Research Foundation Centre of Excellence for Biomedical Tuberculosis Research, Stellenbosch University, Cape Town, South Africa; m South African Medical Research Council, Centre for Tuberculosis Research, Division of Molecular Biology and Human Genetics, Faculty of Medicine and Health Sciences, Stellenbosch University, Cape Town, South Africa; n International Tuberculosis Research Center, Seoul, South Korea; a The Center for Infectious Disease Research, Seattle, WA, USA; b Department of Science and Technology, National Research Foundation Centre of Excellence for Biomedical Tuberculosis Research, Stellenbosch University, Cape Town, South Africa; c South African Medical Research Council, Centre for Tuberculosis Research, Division of Molecular Biology and Human Genetics, Faculty of Medicine and Health Sciences, Stellenbosch University, Cape Town, South Africa; d Mater Medical Research Institute, The University of Queensland, Brisbane, Australia; e Center for Emerging Pathogens, Department of Medicine, Rutgers New Jersey Medical School, Rutgers Biomedical & Health Sciences, Newark, NJ, USA; f Tuberculosis Research Section, Laboratory of Clinical Infectious Diseases, Division of Intramural Research, National Institute of Allergy and Infectious Diseases, National Institutes of Health, Bethesda, MD, USA; g Institute of Infectious Disease and Molecular Medicine, Department of Clinical Laboratory Sciences, University of Cape Town, Cape Town, South Africa; h Western Cape Academic Positron Emission Tomography–Computed Tomography Centre, Tygerberg Academic Hospital, Cape Town, South Africa; i Division of Nuclear Medicine, Department of Medical Imaging and Clinical Oncology, Faculty of Medicine and Health Sciences, Stellenbosch University, Cape Town, South Africa; j South African Tuberculosis Vaccine Initiative, Institute of Infectious Disease and Molecular Medicine & Division of Immunology, Department of Pathology, University of Cape Town, Cape Town, South Africa; k Catalysis Foundation for Health, Emeryville, CA, USA

**Keywords:** Tuberculosis treatment, Host response, Transcriptome, Biomarkers, Mitochondria

## Abstract

Biomarkers for tuberculosis treatment outcome will assist in guiding individualized treatment and evaluation of new therapies. To identify candidate biomarkers, RNA sequencing of whole blood from a well-characterized TB treatment cohort was performed. Application of a validated transcriptional correlate of risk for TB revealed symmetry in host gene expression during progression from latent TB infection to active TB disease and resolution of disease during treatment, including return to control levels after drug therapy. The symmetry was also seen in a TB disease signature, constructed from the TB treatment cohort, that also functioned as a strong correlate of risk. Both signatures identified patients at risk of treatment failure 1–4 weeks after start of therapy. Further mining of the transcriptomes revealed an association between treatment failure and suppressed expression of mitochondrial genes before treatment initiation, leading to development of a novel baseline (pre-treatment) signature of treatment failure. These novel host responses to TB treatment were integrated into a five-gene real-time PCR-based signature that captures the clinically relevant responses to TB treatment and provides a convenient platform for stratifying patients according to their risk of treatment failure. Furthermore, this 5-gene signature is shown to correlate with the pulmonary inflammatory state (as measured by PET-CT) and can complement sputum-based Gene Xpert for patient stratification, providing a rapid and accurate alternative to current methods.

## 1 Introduction

Treatment of pulmonary tuberculosis (TB) disease typically entails a 6-month chemotherapeutic regimen of multiple drugs that provides bacteriologic cure in most patients. However, unfavorable outcomes, such as treatment failure or disease recurrence after discontinuation of chemotherapy, occur even when treating drug-sensitive TB. New technologies are needed to monitor treatment in individual patients and to adapt treatment duration and/or regimens to improve outcomes. This is exemplified by the highly heterogeneous nature of disease pathology, which was clearly illustrated by a recent positron emission tomography and computerized tomography (PET-CT) imaging study of TB patients [1]. This study identified PET-CT patterns similar to active disease in most bacteriologically cured individuals at the end of treatment, and an association between non-resolved lesions and detection of *Mycobacterium tuberculosis* (Mtb) RNA in sputum or lavage samples. Many cured individuals developed new lesions during treatment, and 68% of cured individuals had persistent pulmonary inflammation even one year after the completion of therapy. These results suggest that Mtb is not eradicated in a subset of treated patients, and that non-eradication of Mtb during treatment may pose a risk factor for TB recurrence. While PET-CT itself is not a technology that could be broadly applied as a biomarker, the stratification within bacteriologically cured patients revealed by PET-CT motivates development of novel biomarker technologies that can affordably identify these important subpopulations.

Host blood RNA profiling has shown promise as a TB disease diagnostic [2], [3], [4], [5], [6] and for generating a correlate of risk (the “ACS COR”) that can predict progression from latent to active disease, with 66% sensitivity and 81% specificity within a year of TB diagnosis [7]. Blood profiling can confirm TB at diagnosis and monitor the overall response to treatment [2], [7], [8], [9], but whether these responses are predictive of differential treatment outcomes has not been investigated. Furthermore, it is unknown whether the blood transcriptional signatures for TB provide information that is redundant to established sputum-based assays, such as Gene Xpert cycle threshold (Ct) values [10], or whether complementary analysis of the host and pathogen during TB treatment will yield the most accurate prediction of treatment response.

In this study, a comprehensive whole blood transcriptomic analysis of a well-characterized TB treatment cohort (the Catalysis treatment response cohort, “CTRC”) was performed to identify candidate biomarkers for clinically relevant responses. Previous analyses of this cohort evaluated and identified a variety of potential diagnostic, predictive, and end of treatment biomarkers [1], [10].

## 2 Materials and methods

### 2.1 Clinical definitions and TGAI score

Ethical approval was obtained from the Stellenbosch University Human Research Ethics Committee (registration number N10/01/013). In total, 131 HIV-uninfected adults with newly diagnosed pulmonary TB, as confirmed by sputum culture, were recruited after informed consent between April 2010 and April 2013 at primary healthcare clinics in Cape Town. Gene Xpert Cts were recorded for the majority (but not all) of patients at each time point. Patients' clinical outcomes were classified as ‘cured’ if they proved and maintained sputum culture negativity by month 6 after treatment initiation (M6), ‘failed’ if the M6 culture was still positive, and ‘un-evaluable’ if contamination caused uncertainty in outcome. We note that none of the treatment failures achieved culture negativity at any time point during treatment. Rapid responders are defined as those patients with a negative bacterial culture at or before week 4, followed by no reversion to positive culture throughout the course of treatment. Total Glycolytic Activity Index (TGAI) is a product of lesion volume and FDG uptake intensity and represents the total inflammatory burden. TGAI was measured at every time point in all the lesions throughout the lungs. After a volume of interest for the lungs was created, the lesions were delineated using an adaptive-threshold technique based on the background uptake in lesion free lung tissue. The uptake intensities were also standardized based on background uptake. Further detail on the PET-CT analysis is provided in Ref. [1].

### 2.2 RNA processing and sequencing

Whole blood was collected from patients in the CTRC directly into PAXgene blood RNA tubes (PreAnalytiX, Hombrechtikon, Switzerland), which were stored at −20 °C. RNA was extracted from PAXgene tubes and globin transcript depletion (GlobinClear, ThermoFisher Scientific, MA, USA) was followed by cDNA library preparation using the Illumina TruSeq Stranded mRNA sample preparation kit (Illumina, CA, USA). Globin transcript depletion, cDNA library preparation and RNA sequencing were performed by Beijing Genomics Institute (Shenzhen, China). The sequencing strategy was 70 million 50bp paired-end reads per sample, and sequencing was performed on Illumina (San Diego, CA) HiSeq-2000 sequencers. FASTQ sequences were transferred to the Center for Infectious Disease Research for analysis.

### 2.3 Quality control and processing of RNA-Seq data

Read pairs were preprocessed using in-house scripts that adjust base calls with phred scores <5 to ‘N’ and remove read pairs for which either end has fewer than 30 unambiguous base calls, a method that also indirectly also removes pairs containing mostly adaptor sequences. Read pairs were aligned to the human genome (hg19) using STAR (v2.3.1d) [11], taking as input the Ensembl GRCh37.74 splice junction table and allowing novel splice junction detection. Junction-level expression values were standardized for each sample using a set of reference features, such that:

$$abundance_{j}=\log_{2}\left(counts_{j}+1\right)-\sum_{r\in refs}\frac{\log_{2}\left(counts_{r}+1\right)}{N_{refs}}$$

Mapped read pairs were assigned to genes by collapsing all transcripts into a single gene model and then counting the number of reads that fully overlap the resulting exons using htseq (v. 0.6.0) [12] with strict intersection and including strand information. Gene models for protein-coding genes were downloaded Ensembl (GRCh37.74). Reads that mapped to multiple locations were only counted once and those mapping to ambiguous regions were excluded. Log2-transformed values of counts normalized by adjusted library counts were computed using the cpm function of the edgeR package [13]. RNA-seq signatures were further confirmed via qRT-PCR, ensuring accuracy of the observed signals.

### 2.4 Datasets and transcriptomic signatures

The ACS COR employed in this manuscript is an RNA-seq based signature that was constructed to predict which individuals with a latent TB infection are at risk of progressing to active disease using samples from the Adolescent Cohort Study (ACS) [7]. The ACS comprises samples taken longitudinally from 153 adolescents from the Worcester region of South Africa. Over the course of the study, 46 of the adolescents developed active TB disease (referred to as ‘Progressors’), while the remaining 107 adolescents remained healthy (referred to as ‘Non-progressors’). The ACS COR was developed to discriminate progressors from non-progressors prior to the diagnosis of disease, and is an ensemble of 258 pairwise models featuring 63 unique splice junctions from 16 unique genes. When presented with a sample, each pairwise model casts a binary case/control vote indicating whether the person is at risk of progressing or not. The overall ACS COR score is the percentage of ‘case’ votes. See Ref. [7] for full details.

The DISEASE and FAILURE signatures were generated using the Well-Fit Pairs algorithm and the RESPONSE5 signature was constructed using the Pair Ratio approach. Both of these algorithms are generalizations of the pairwise approach developed in Ref. [7] to generate the ACS COR signature. Full details of these algorithms and construction of the signatures are provided in the *Supplementary Materials and Methods*. In brief, the DISEASE and FAILURE signatures were developed to discriminate between two well-defined groups of samples, referred to generically as ‘cases’ and ‘controls’. For the DISEASE signature, the ‘cases’ were samples taken from cured patients at pre-treatment, and the ‘controls’ were samples taken from the same patients at week 24. For the FAILURE signature, the ‘cases’ were samples taken from treatment failures at pre-treatment, and the ‘controls’ were samples taken from cured patients at pre-treatment. We then performed a univariate feature selection, selecting all splice junctions that strongly differentiated the cases from the controls (p < 10^−20^ for DISEASE, p < 0.01 for FAILURE). A resampling approach was used for the FAILURE signature due to the imbalance in number of treatment failures and cured patients. After univariate feature selection, a pairwise selection step was performed, looking for pairs of splice junctions that provide complementary information and are best able to differentiate the cases and controls. All pairs with both sensitivity and specificity above 80% were selected. For each of the selected pairs, a binary threshold was established that maximizes the sum of sensitivity and specificity on the training set. The ensemble of selected pairs was then applied to previously unseen samples. Each pair makes a ‘case’ or ‘control’ vote, and the final score is the percentage of all pairs that voted ‘case’. The values for the cutoffs during both the univariate and pairwise selection steps were optimized using leave-one-out cross-validation. Consult the *Supplementary Materials and Methods* for full detail.

### 2.5 Statistical analysis

The ability of a given signature score to discriminate two groups was quantified via the Wilcoxon U statistic (in the form of an ROC AUC) and its associated p-value. When signatures that were trained to make one type of discrimination are applied to make cross-predictions involving a second type of discrimination, there is an *a priori* expectation that the scores will be higher in one group rather than the other. For example, when the correlate of risk for progression to TB disease (ACS COR) is used to predict treatment failure, it is expected that treatment failures will better resemble at-risk individuals than controls, and will therefore have higher scores. Thus, when signatures are used to make cross-predictions, one-sided p-values are reported. Outlier TB patients with exceptionally low ACS COR scores at pre-treatment baseline were defined as having scores more than 1.5 times the interquartile range below the lower quartile. Significant enrichment of sample or gene groups (including the modular enrichment analysis) was calculated using the hypergeometric distribution and associated p-value. Differential expression of genes was determined using the Wilcoxon U statistic. P-values were adjusted for multiple hypothesis testing using the False Discovery Rate (FDR) method of Benjamini-Hochberg [14] and significance thresholds of FDR < 0.3 and nominal p < 0.01 were employed applied. The extent to which RESPONSE5 scores are able to complement Gene Xpert Ct in stratifying TB treatment patients was assessed using logistic regression. A logistic regression model was fit as CLASS ∼ Xpert (where CLASS is a binary labeling of the two groups being compared) and compared to a model incorporating the RESPONSE5 score, CLASS ∼ Xpert + RESPONSE5. The p-value for the increase in fit afforded by adding in RESPONSE5 was computed via a chi squared test using the R function anova() [15].

## 3 Results

### 3.1 RNA-sequencing of the CTRC

The CTRC consists of HIV-negative adult patients from South Africa who were diagnosed with pulmonary TB and underwent standard treatment [1], [10], [16]. Whole blood transcriptomes of 99 patients were measured via RNA-sequencing (RNA-seq) prior to the start of treatment and at one, four, and 24 weeks after treatment initiation and were compared to transcriptomes from 29 healthy controls. 84 patients met or exceeded the WHO definition for cure after the standard six-month treatment (“cures”), 8 patients did not achieve bacteriological cure (“treatment failures”), and the remaining seven patients were possible cures or unevaluable (not analyzed in this study). Of the 8 treatment failures, only one was determined to be infected with MDR TB. Samples were not available from all patients at every time point. The number of samples available at each time point for each group of patients is provided in Table S1. For full clinical definitions and demographic detail, see *Materials and Methods* and [1].

The cured patients could be further stratified according to the rate of bacteriological conversion or the extent of pulmonary inflammation at the end of treatment. Of the 84 cures, 18 achieved bacteriological conversion within 4 weeks and were considered “rapid converters.” Furthermore, as previously reported [1], PET-CT analysis revealed that a minority of bacteriologically cured patients exhibited full resolution of pulmonary inflammation (defined as an absence of increased [18F]FDG uptake as compared to surrounding healthy tissue) at the end of treatment (“EOT”). Of the 84 cures, 11 were considered “resolved” at EOT according to these criteria, while the remainder are considered “unresolved”.

### 3.2 Symmetry between TB disease progression and resolution during treatment

**Fig. 1:**
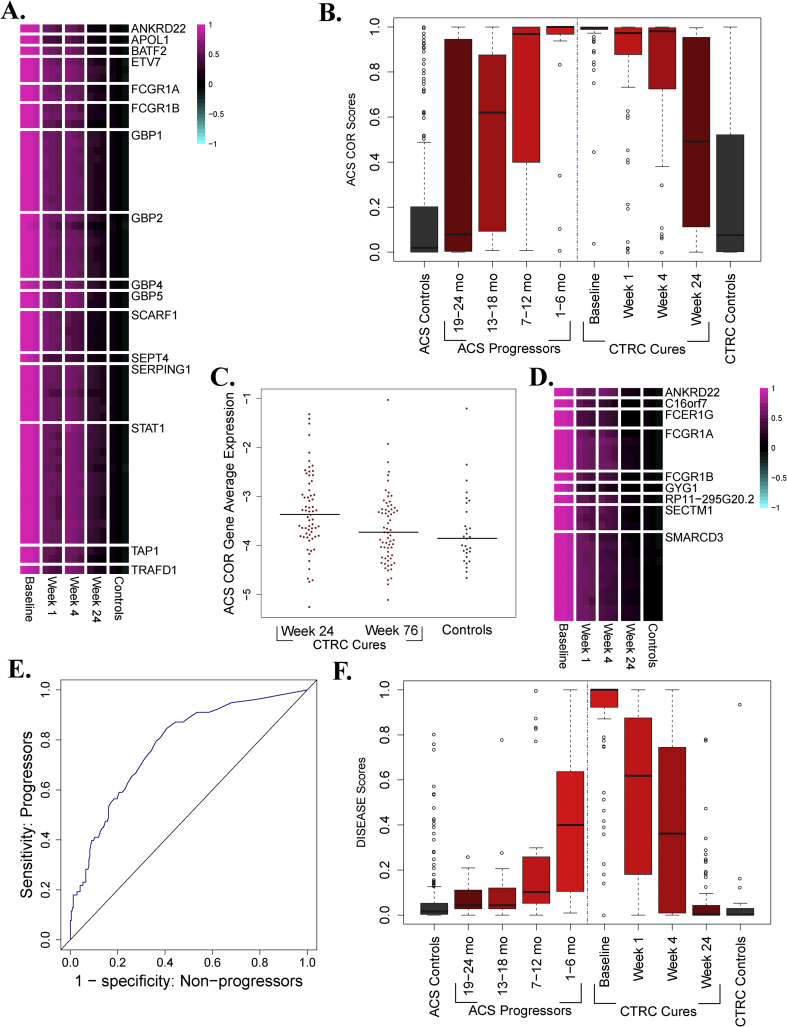
**Symmetry between progression and treatment response**. **A**. The expression of each junction in the ACS COR at each time point and in controls. For each group, the middle column is the mean and the right/left columns are the mean ± SEM. Multiple junctions from the same gene are indicated by multiple rows. **B**. ACS COR scores on samples from the ACS (left) and CTRC (right) datasets. After treatment initiation, scores significantly decrease from baseline by week 1 (Wilcoxon p < 10^−5^) and remain significantly higher at the end of treatment compared to controls (Wilcoxon p = 0.001). **C**. Average expression of ACS COR genes as measured by qRT-PCR. Expression is significantly higher in cures at the end of treatment than in controls (Wilcoxon p = 0.003) but is no longer significantly higher one year after the end of treatment (Wilcoxon p = 0.31). **D**. The expression of each junction in the DISEASE signature at each time point and in controls. For each group, the middle column is the mean and the right/left columns are the mean ± SEM. Multiple junctions from the same gene are indicated by multiple rows. **E**. ROC curve for the DISEASE signature predicting TB progression on the ACS dataset. AUC = 0.78, p < 10^−13^. **F**. DISEASE scores on samples from the ACS (left) and CTRC (right) datasets. For the heatmaps in Figs. 1A and 1D, the junction-level mean ± standard error of the mean is plotted for each group of samples. Values are shifted such that the mean expression in controls is set to zero, and scaled such that the values lie between −1 and 1. The junctions plotted and reference junctions used for standardization are given in Tables S7 and S8.

Previously, a transcriptomic analysis of the Adolescent Cohort Study (“ACS”) TB progression cohort [17] yielded a whole blood transcriptomic signature that predicts risk of TB disease progression (the “ACS COR” [7]). Application of the ACS COR to published microarray data [8] suggested that ACS COR scores return to healthy control levels after standard TB treatment. To confirm this behavior, the ACS COR was applied to the CTRC. In patients that achieved microbiological cure, expression of the genes within the ACS COR was highly elevated compared to controls prior to treatment initiation and rapidly declined upon the start of therapy (Fig. 1A). Comparing ACS COR scores for CTRC cures to scores from the ACS TB progression cohort [7] revealed a symmetrical pattern (Fig. 1B). While ACS COR scores increased with proximity to TB diagnosis in individuals who are progressing to active TB disease (left half of Fig. 1B), scores decreased significantly after only one week of treatment (p < 10^−5^) in cures from the CTRC and continued decreasing over the entire treatment course (right half of Fig. 1B), although no decrease was observed between week 1 and week 4. Despite significant decreases over the course of treatment, ACS COR scores and the expression of ACS COR genes were significantly higher in CTRC cures compared to healthy controls at every time point during the course of treatment, including after the completion of drug therapy at week 24 (Fig. 1C and Table S2).

Further inspection of the data revealed that while pre-treatment ACS COR scores were maximally saturated for most patients that would be cured, a small number of patients (15/78) were outliers and had comparatively lower baseline scores (Fig. 1B). Comparing the bacteriological conversion rates of these patients with low baseline ACS COR scores to the overall cured population revealed a two-fold enrichment of rapid converters, an association that was statistically significant (p = 0.02). Direct prediction of rapid response using the ACS COR scores confirmed that low baseline expression of the ACS COR is predictive for rapid successful treatment (p = 0.002, Table S2).

To further investigate the symmetry between TB risk and resolution, a signature that optimally monitors the treatment response in CTRC cures was applied to predict TB progression in the ACS cohort. Using the RNA-seq transcriptomes from the CTRC, the “DISEASE” signature was constructed to discriminate baseline from week 24 gene expression in cures. The DISEASE signature consists of 26 splice junctions (Fig. 1D) from 9 genes (3 of which are in the ACS COR) that are maximally expressed at baseline and decline during treatment. When applied to the ACS cohort, the DISEASE signature predicted TB progression with accuracy comparable to the ACS COR itself (AUC = 0.78, p < 10^−13^; Fig. 1E). DISEASE scores exhibited a temporal pattern similar to ACS COR (Fig. 1F), increasing with proximity to TB diagnosis and decreasing over the course of treatment in cured patients, although some differences between The ACS COR and DISEASE score patterns are present. Most notably, DISEASE scores in CTRC cures at week 24 are not significantly higher than in controls, unlike ACS COR scores. This difference arises because the DISEASE signature was constructed to include genes that exhibit maximal changes over the course of treatment and therefore includes mostly genes that have returned to control levels by week 24. Taken together, the results from the ACS COR and DISEASE signatures present compelling evidence for symmetry between progression to active disease and resolution of disease during drug therapy.

### 3.3 Host blood RNA signatures predict treatment failure

**Fig. 2:**
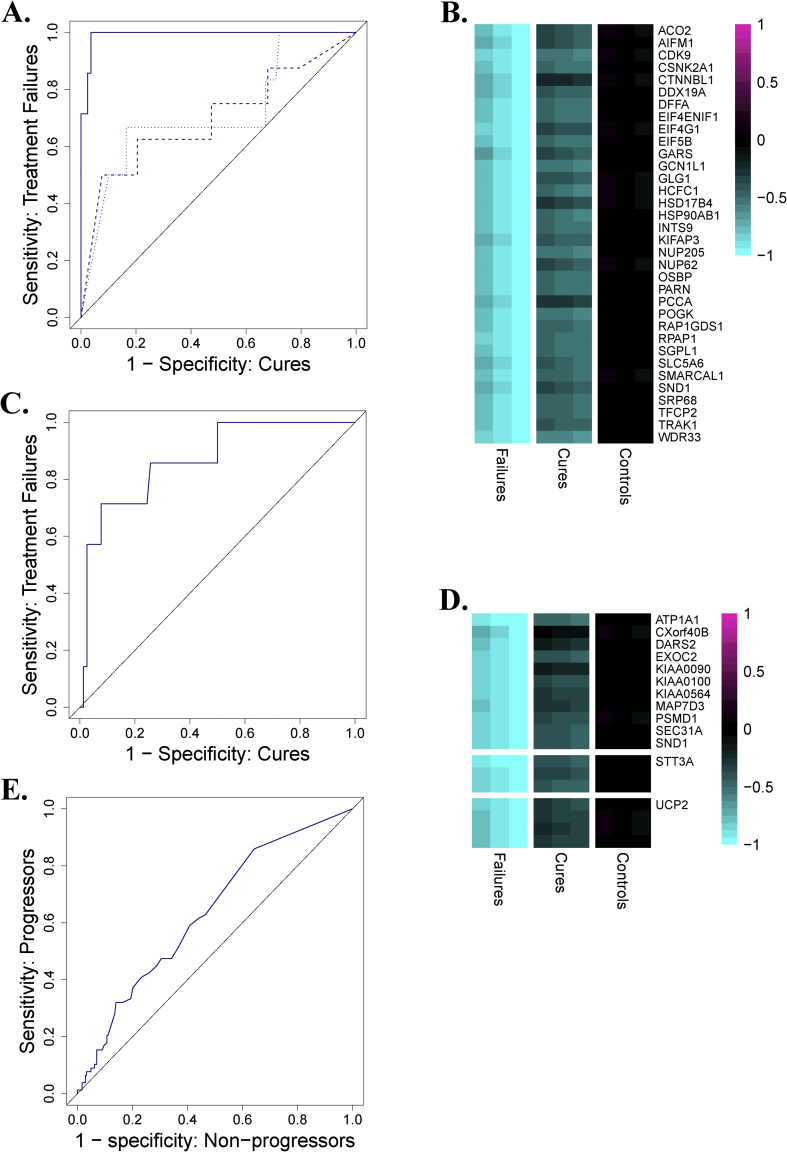
**Prediction of treatment failures**. **A**. DISEASE score ROCs for predicting treatment failure using gene expression profiles at week 24 (solid; AUC = 0.99, p = 0.000003), week 4 (dashed; AUC = 0.70, p = 0.03) and week 1 (dotted; AUC = 0.72, p = 0.04). **B**. Pre-treatment expression of mitochondria genes in treatment failures, cures and controls. For each group, the middle column is the mean and the right/left columns are the mean ± SEM. **C**. Leave-one-out cross-validation ROC curve for FAILURE signature (AUC = 0.87, p = 0.0006), demonstrating the ability to predict treatment failure using pre-treatment expression profiles. **D**. Pre-treatment expression of the junctions in the FAILURE signature in treatment failures, cures and controls. For each group, the middle column is the mean and the right/left columns are the mean ± SEM. Multiple junctions from the same gene are indicated by multiple rows. **E**. ROC curve of the FAILURE signature predicting progression on the ACS dataset (AUC = 0.63, p = 0.0001). For the heatmaps in Figs. 2B and D, junction (2B) or gene (2D) -level mean ± standard error of the mean is plotted for each group of samples. Values are shifted such that the mean expression in controls is set to zero, and scaled such that the values lie between −1 and 1. The junctions plotted and reference junctions used for standardization are given in Table S9.

Within the CTRC, 8 patients did not achieve microbiological cure. To determine whether adherence to therapy was predictive of treatment failure, we used logistic regression to model failure as a function of the total cumulative number of missed doses over the 6-month treatment regimen. This analysis revealed a statistically significant association between missed doses and failure (p = 0.006), but not a perfect association (ROC AUC = 0.78, optimal operating point = 70% sensitivity at 90% specificity), as there were adherent treatment failures and non-adherent cures. We also tested whether the blood RNA-based DISEASE and ACS COR signatures could discriminate these treatment failures from the successful cures at each time point. The DISEASE signature significantly identified the onset of failure more than five months before the failures were clinically identified (week 1 and week 4 ROC AUCs = 0.70-0.72, p < 0.05; Fig. 2A), and nearly perfectly discriminated failures from cures at the end of treatment (AUC = 0.99, p < 10^−5^; Fig. 2A), demonstrating that treatment failures have not achieved the same return to homeostasis that the cures have by week 24. The ACS COR also strongly discriminated treatment failures from cures at week 24 (AUC = 0.95, p = 0.00005; Fig. S1) and significantly predicted treatment failure at week 4 (AUC = 0.72, p = 0.02; Fig. S1). The ability of DISEASE and ACS COR signatures to prospectively identify patients at risk of treatment failure demonstrates that the genes undergoing the most substantial changes during successful treatment are changing to a smaller degree in treatment failures and that this difference is apparent after only one week of treatment. Although treatment failure was associated with non-adherence, the ability of blood RNA signatures to identify patients at risk of failure using measurements from intermediate time points is potentially clinically useful given that it is an objective molecular readout that does not depend on self-reporting.

While the ability to predict treatment failure after only 1 or 4 weeks of therapy would provide a valuable tool to triage patients at risk of failure toward an enhanced treatment regimen and more frequent monitoring, prediction of treatment failure prior to the start of treatment would also be of considerable value, both for treatment modification and for insight into host response deficiencies that may complicate treatment success. We therefore evaluated whether treatment failures could be discriminated from cures in the CTRC based on their pre-treatment gene expression profiles. 474 genes were significantly differentially-expressed between treatment failures and cures at baseline, 47 of which were higher in the treatment failures and 427 of which were higher in the cures (Table S3). The set of genes that was higher in failures was enriched for genes that decrease significantly over the course of successful treatment (37/47 genes, p < 10^−25^), and the set of genes that was higher in cures was enriched for genes that increase significantly over the course of successful treatment (397/427 genes, p < 10^−68^). Enrichment analysis [18], [19], [20] revealed that the module ‘enriched in neutrophils’ is significantly over-represented in the gene set that is expressed higher in treatment failures at baseline (p < 0.03, FDR < 0.08; Table S4). This suggests the presence of a state of enhanced inflammation prior to treatment in patients who will ultimately not be cured. Conversely, the set of genes that are higher in cures at baseline were enriched for the modules ‘mitochondria’, ‘unfolded protein response’ and ‘nuclear pore complex’ (p < 0.007, FDR < 0.09; Table S4). The strongest enrichment in treatment failures was pre-treatment suppression of mitochondrial gene expression (Fig. 2B, p < 10^−8^, FDR <10^−6^).

The existence of a large set of differentially-expressed genes with statistically significant biological enrichments indicated that prediction of treatment failure using pre-treatment gene expression profiles may be possible. We therefore employed machine learning techniques to develop a signature of treatment failure using splice junction counts at pre-treatment baseline. Leave-one-out cross-validation analysis indicated strong performance for a baseline signature of treatment failure (ROC AUC = 0.87, p = 0.0006; Fig. 2C). These results motivated the development of the “FAILURE”, which consists of 18 splice junctions from 13 genes that are expressed at a lower levels in treatment failures than in cures at pre-treatment baseline (Fig. 2D). Interestingly, none of the genes in the FAILURE signature had been implicated by any other previously reported transcriptional signature of TB disease pathology, indicating a new association between biological pathways and treatment outcome. Importantly, the FAILURE signature significantly predicted TB disease progression when evaluated using the ACS dataset (AUC = 0.63, p = 0.0001; Fig. 2E), albeit less accurately than the ACS COR or DISEASE signatures. The fact that FAILURE is able to significantly predict TB disease risk in an independent cohort confirms that these genes are regulated during TB pathology and suggests that they may represent a minor immune response in terms of disease progression but make a critical contribution to successful treatment.

As mentioned above, the cumulative number of missed doses was predictive of failure, suggesting that adherence was a major driver of treatment failure in the CTRC, and our identification of a pre-treatment FAILURE signature appears at odds with this result. However, the etiology of non-adherence to treatment is unclear, as the nature of the infection and severity of disease may influence adherence itself. For example, patients with severe disease may not experience a sufficiently beneficial response to therapy that justifies the side effects. Similarly, patients that are pre-disposed to non-adherence may wait longer before seeking treatment and may thereby develop more severe and harder to treat disease. To reconcile this apparent contradiction, we performed logistic regression analyses comparing models based on the number of cumulative missed doses and pre-treatment levels of the FAILURE signature, individually and in combination. While the FAILURE signature significantly complemented the cumulative number of missed doses for predictive treatment failure (p = 8 × 10^−8^), the cumulative number of missed doses did not complement the FAILURE signature (p = 0.999). This result suggests that the FAILURE signature captures pre-existing biological differences between the disease states of patients that will become cured and those that will fail that may influence outcome either by indirectly influencing treatment adherence (for reasons described above) or through biological processes that are irrespective of adherence.

### 3.4 A parsimonious 5-gene signature for TB treatment monitoring and predicting failure

**Fig. 3:**
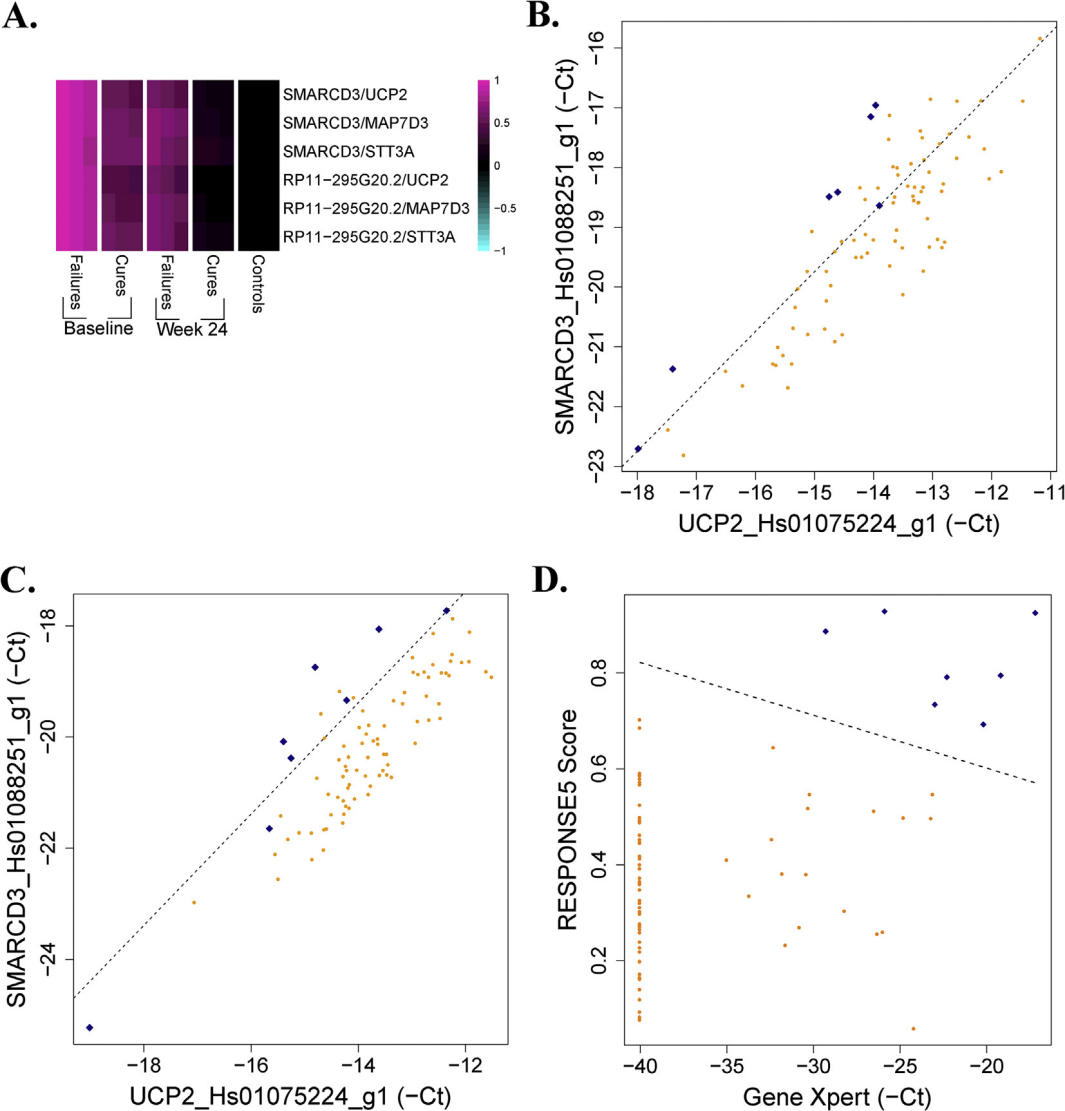
**The RESPONSE5 signature**. **A**. The RESPONSE5 signature involves six ratios of assays, each involving one assay from the DISEASE signature and one from the FAILURE signature. Ratios of expression for the pairs of assays in the RESPONSE5 signature are plotted for treatment failures and cures, at baseline and week 24, and for controls. For each group, the middle column is the mean and the right/left columns are the mean ± SEM. **B**. Pre-treatment Cts of SMARCD3 and UCP2 discriminate treatment failures (blue diamonds) from cures (orange circles). Negative Cts are plotted throughout, such that increasing values correspond to increasing expression. **C**. Week 24 Cts of SMARCD3 and UCP2 discriminate treatment failures (blue diamonds) from cures (orange circles). **D**. Week 24 RESPONSE5 scores and Gene Xpert Cts perfectly discriminate treatment failures (blue diamonds) from cures (orange circles). (For interpretation of the references to colour in this figure legend, the reader is referred to the web version of this article.)

The DISEASE and FAILURE signatures each capture salient aspects of the response to TB treatment (Table S2). For application in future clinical studies of TB treatment, a compact and easily implementable signature that simultaneously offers all of the strengths of the DISEASE and FAILURE signatures is desirable. To accomplish this, both signatures were adapted to and confirmed on the qRT-PCR platform (*Supplementary Materials and Methods*). The assays composing the signatures were then pooled and mined to systematically identify small gene subsets that simultaneously discriminate baseline from week 24 expression among and between cures and treatment failures from cures using baseline gene expression. The pair of assays that best simultaneously achieves these discriminations was first identified, and then the assays that lead to the greatest increase in overall fit were sequentially added until no further appreciable increases were achieved (Table S5). The resulting five-assay signature (“RESPONSE5”) consists of six pairs of assays, each pair involving one assay from the DISEASE signature and one from the FAILURE signature (Fig. 3A). The ability of raw Cts from the best individual pair in the signature, involving the genes SMARCD3 and UCP2, to simultaneously discriminate treatment failures from cures using both pre-treatment and week 24 gene expression profiles is demonstrated in Fig. 3B and C. The RESPONSE5 signature is formulated in terms of qRT-PCR Cts and requires the measurement of only five assays. It could therefore provide the basis of an easily implementable and affordable clinical test for TB treatment outcome.

The RESPONSE5 signature implicates a long non-coding RNA (lncRNA), RP11-295G20.2., previously unassociated with TB pathology. Example RNA-seq alignments for the gene are given in Fig. S2A. This gene exhibits enormous changes over the course of treatment and is higher in treatment failures than cures at week 24 and at baseline, as measured by both RNA-seq and qRT-PCR (Table S6). Interestingly, RP11-295G20.2 remains significantly higher in cures one year after the end of treatment than in controls, in contrast to the genes in the ACS COR (p = 0.01; Fig. S2B). RP11-295G20.2 is also up-regulated during progression to active TB disease in the ACS dataset (Fig. S2C).

### 3.5 RESPONSE5 complements Gene Xpert for predicting treatment outcomes

Previous analysis of this cohort demonstrated that sputum-based Gene Xpert Ct values (Gene Xpert), a widely-implemented measurement of bacterial load in clinical studies of TB treatment, are predictive of treatment responses [10], and a previous study of a separate cohort showed similar ability of Gene Xpert Cts to discriminate treatment failures at the end of treatment [21]. In order for a transcriptomic signature to have novel utility, it must provide value beyond that of Gene Xpert Cts alone. We tested whether combining bacterial (Gene Xpert Ct) and host (blood RNA signatures) measurements would result in more accurate prediction of outcome than either measurement alone. To test this hypothesis, we assessed the extent to which the RESPONSE5 signature can lead to significant improvements in prediction accuracy when combined with Gene Xpert Cts.

RESPONSE5 significantly complemented the ability of Gene Xpert Cts for predicting treatment failure at all time points (p < 0.02, Table 1), including at week 24, where a combination of Xpert Ct and RESPONSE5 was able to classify treatment failures and cures with 100% accuracy (Fig. 3D). The culture-based tests currently used for determining successful treatment require up to six weeks to generate results. The combination of RESPONSE5 and Gene Xpert could provide a rapid and specific alternative to measuring clinical outcome at the end of standard therapy.

**Table 1:** RESPONSE5 significantly complements Gene Xpert Ct values for predicting treatment failure.

Treatment Failures vs Cures:	Xpert Alone		Xpert + RESPONSE5		Improvement of fit: p
	Sensitivity	Specificity	Sensitivity	Specificity	
Baseline	83%	69%	83%	99%	0.00052
Week 1	100%	26%	100%	60%	0.015
Week 4	100%	40%	83%	97%	0.0015
Week 24	100%	88%	100%	100%	0.000018

### 3.6 RESPONSE5 tracks PET-CT-based measurements of pulmonary TB disease

**Fig. 4:**
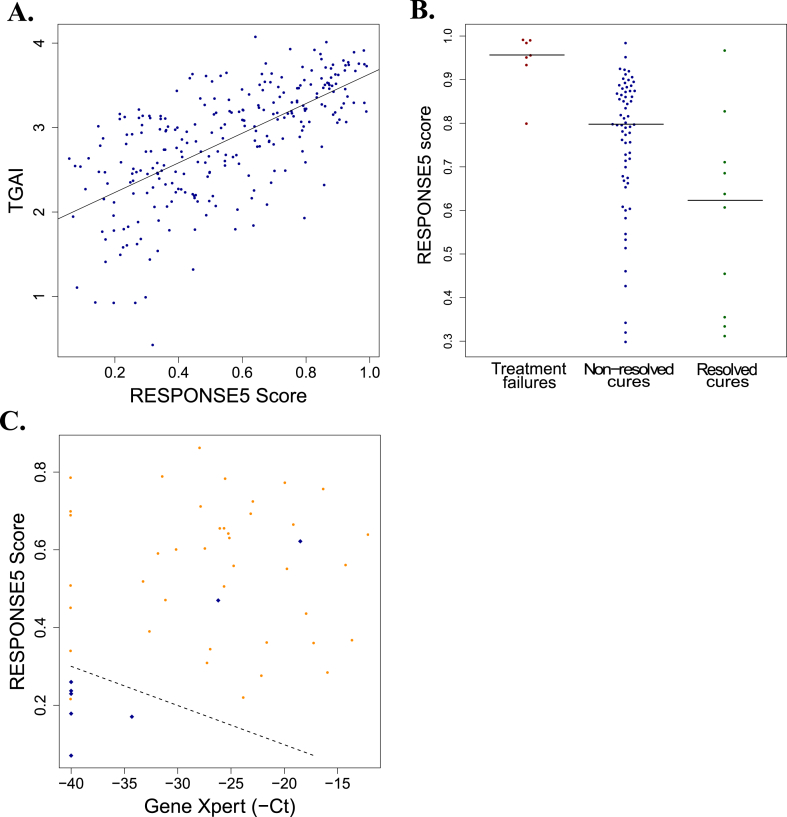
**The RESPONSE5 signature and PET-CT stratification**. **A**. RESPONSE5 scores plotted versus TGAI for all available samples in the CTRC (Spearman ρ = 0.68, p < 10^−37^). The solid line is the linear regression fit to the data. **B**. Pre-treatment RESPONSE5 scores stratify patients according to ultimate treatment outcome. Treatment failures vs cures: AUC = 0.92, p = 0.00015. Non-resolved vs resolved cures: AUC = 0.74, p = 0.0074. **C**. Week 4 RESPONSE5 scores and Gene Xpert Cts discriminate PET-CT resolved cures (blue diamonds) from PET-CT unresolved cures (orange circles). (For interpretation of the references to colour in this figure legend, the reader is referred to the web version of this article.)

PET-CT scans were administered to the patients in the CTRC and analyzed to characterize the extent of pulmonary inflammation before and during TB treatment [1]. The total glycolytic activity index (TGAI) was introduced as a quantification of pulmonary inflammation (*Materials and Methods*). RESPONSE5 scores correlated strongly with TGAI (Spearman ρ = 0.68, p < 10^−37^; Fig. 4A), indicating that the RESPONSE5 signature is accurately reflecting TB-associated pulmonary inflammation. In Ref. [1], it was found that the majority of microbiological cures had residual active lesions in their lungs according to PET-CT, even at the end of treatment. Patients were stratified into those who had resolved all lesions by week 24 and those who hadn't. The RESPONSE5 signature predicted week 24 PET-CT status at baseline, week 1 and week 4 (AUC = 0.72-0.74, p < 0.02; Table S2). This stratification is illustrated in Fig. 4B, where it is shown that the RESPONSE5 signature stratifies all patients according to treatment outcome – failure, cure with non-resolved lesions, or cure with full resolution of all lesions – using only baseline gene expression profiles. This result suggests that RESPONSE5 is capturing aspects of the host state that play crucial roles in determining ultimate treatment outcome, not just in terms of culture negativity, but also in terms of resolution of inflammation. The ability to prospectively predict PET-CT resolution is further demonstrated in Fig. 4C, where Gene Xpert and RESPONSE5 provide a highly specific predictor of PET-CT resolution at week 4. Many patients have high Gene Xpert Ct values at week 4, but only those who simultaneously have low RESPONSE5 scores are likely to fully resolve all lung inflammation by the end of treatment. Lastly, the DISEASE signature also tracked PET-CT-based measures of pulmonary inflammation and significantly predicted week 24 PET-CT status using gene expression measurements at all time points, including pre-treatment baseline (AUCs = 0.70-0.77, p < 0.02; Fig. S3).

## 4 Discussion

In this manuscript, the ability of four different host blood RNA signatures to capture differential responses to TB treatment has been assessed. The performance of all four signatures on all of the comparisons discussed in the manuscript is summarized in Table S2.

The ACS COR signature was derived entirely from a dataset independent of the CTRC and was constructed to predict TB disease progression. ACS COR scores were elevated in TB patients relative to controls before treatment begins and decrease over the course of successful treatment, although scores are still significantly higher in cured patients at week 24 than in healthy controls. This result is consistent with the PET-CT-based observation that most microbiologically cured patients exhibit persistent pulmonary inflammation at the end of treatment [1]. However, one year after completion of treatment, 67% of patients had improved PET-CT scans and, similarly, the average expression of ACS COR genes returned to control levels within that timespan (Fig. 1C). ACS COR scores were highly elevated in treatment failures at week 24 compared to cures and there was a monotonic increase in the ability of the ACS COR to identify treatment failures over time. The ACS COR was also able to stratify the cures, both in terms of conversion rate and in terms of PET-CT status at end of treatment (Table S2). The significant predictive performance of the ACS COR on the CTRC validates the hypothesis that the biological processes underlying progression and resolution of TB are related.

The DISEASE signature was built from the CTRC dataset to identify the genes that exhibited the greatest changes over the course of successful treatment using data from cured patients alone. The performance of the DISEASE signature for identifying treatment failure, rapid conversion and PET-CT resolution were similar to that of ACS COR, with stronger and earlier discrimination of treatment failure and PET-CT resolution, and weaker identification of rapid conversion being observed (Table S2). These results demonstrate that the genes that change most over the course of successful treatment are changing at differential rates in treatment failures, rapid responders and resolved cures. The relevance of the DISEASE signature to TB pathology was independently validated by its strong performance in the external ACS TB disease progression dataset (Fig. 1E).

The FAILURE signature was derived from the CTRC to specifically predict treatment failure based on pre-treatment gene expression profiles. A rigorous cross-validation analysis suggested that such prediction is possible, but true confirmation of the signature requires validation on an independent cohort. The genes that maximally discriminate treatment failures from cures at baseline have not previously been implicated in host blood RNA signatures of TB disease pathology, but the FAILURE signature prediction of TB disease progression in the ACS dataset supports a role for these genes in TB pathology. Furthermore, FAILURE scores significantly stratify end-of-treatment PET-CT resolution using pre-treatment baseline gene expression (Table S2), providing strong evidence that the signature identifies a pre-treatment state that is a crucial determinant of overall treatment outcome. What appears to be driving the signature is that mitochondrial genes are highly down-regulated in treatment failures relative to cures at pre-treatment baseline. Evidence has been presented that Mtb targets host mitochondrial function to regulate cell death, and this behavior has been linked to virulence [22], [23], [24], [25]. Combined with the results presented above, this suggests that interactions between Mtb and host mitochondria are a crucial determinant of outcome both during infection and treatment.

The final signature, RESPONSE5, combines the most desirable aspects of the DISEASE and FAILURE signatures into a single small, clinically-implementable qRT-PCR-based signature. RESPONSE5 is formulated in terms of qRT-PCR Ct values, is ratiometric and therefore does not require housekeeping genes, and involves the measurement of only five primer/probe reagents. RESPONSE5 accurately tracks pulmonary inflammation as measured by PET-CT, including discrimination between cured patients that have fully resolved inflammation compared to others. We demonstrated that RESPONSE5 provides complementary information to Gene Xpert (Table 1) and enables perfect discrimination of treatment failures from cures at the end of standard therapy when these measures of host and pathogen are combined. If this result is confirmed in an independent trial, RESPONSE5 and Gene Xpert would provide a fast, inexpensive and clinically-implementable alternative to determining treatment outcome using bacterial culture. As RESPONSE5 was optimized using samples from the CTRC, it remains necessary to confirm prediction performance on independent cohorts.

Of the five genes in RESPONSE5, SMARCD3 is the only one to have appeared in a prior TB blood RNA signature, discriminating active TB disease from healthy controls in Refs. [3], [4], [26] and discriminating TB from other diseases in Ref. [5]. Of the novel genes, UCP2 is highly down-regulated in treatment failures compared to cures at pre-treatment baseline and has been shown to be involved in fatty acid metabolism that promotes NLRP3 inflammasome activation during sepsis [27]. Associations between NLRP3 and control of TB have been indicated by human NLRP3 gene variants that limit intramacrophage growth of Mtb [28] and Mtb-driven activation of the NLRP3 inflammsome [29]. Another novel gene in the signature, STT3A, has been shown to play a crucial role in the signaling of pattern recognition receptors in plants [30], possibly indicating a similar role in animals as has been observed for NLR proteins [31]. The RESPONSE5 signature implicates a long non-coding RNA (lncRNA), RP11-295G20.2., a class of novel transcriptional and post-transcriptional regulators that have been shown to be key mediators of immune response [32], but, to our knowledge, have not yet been shown to play a role in TB pathology.

Modulating the host response is a promising therapeutic approach for pulmonary TB [33]. The novel TB-associated genes identified in this study, particularly baseline suppression of mitochondrial genes as a precursor to failure, offer candidate targets worthy of further investigation, both as biomarkers and as potential avenues to new therapeutics.

In this manuscript, we have presented several host blood RNA signatures and demonstrated that they stratify patients according to their outcome to TB treatment. While the current study is insufficient to conclusively determine the mechanistic underpinnings of the identified signatures and to firmly establish the causal link between the signatures and treatment outcome, the formulation of the signatures allows for direct investigation of the underlying processes in future studies. The ability to stratify patients based on baseline gene expression profiles into groups at high or low risk of failure and recurrence would be a powerful tool, allowing for the personalization and customization of treatment regimens and increased counseling and monitoring for high risk patients. Furthermore, clinical trials would be empowered if drug testing did not suffer from failures that are actually related to failure of a supplemental host response rather than failure of the antibiotic. The results from the CTRC provide compelling evidence for the role host blood RNA biomarkers can play in improving TB treatment efficacy.

## One sentence summary

Clinically-implementable host blood RNA signatures predict tuberculosis treatment outcome.

## Appendix A Supplementary data

Fig. S1 ACS COR scores predict treatment failure. Fig. S1

Fig. S2 RP11-295G20.2. Fig. S2

Fig. S3 DISEASE scores predict PET-CT resolution. Fig. S3

Fig. S4 DISEASE and FAILURE signatures were confirmed via qRT-PCR. Fig. S4
