# Supplementary material for: Host blood RNA signatures predict the outcome of tuberculosis treatment
Source: Tuberculosis (Edinb). 2017 Dec;107:48–58. doi: 10.1016/j.tube.2017.08.004 (PMC5658513; doi:10.1016/j.tube.2017.08.004)
Supplement: Fig. S1 — ACS COR scores predict treatment failure. [file mmc1.docx]

Supplementary Materials:

Supplementary Materials and Methods

Technical descriptions of transcriptomic signatures

This supplement describes in detail the derivation and implementation of the novel signatures DISEASE, FAILURE and RESPONSE5 utilized in the manuscript. We begin by describing the two RNA-seq signatures, DISEASE and FAILURE. Next, we address how these signatures were adapted to qRT-PCR. The third section describes the overall structure of the RESPONSE5 signature, why that structure was selected, and how to compute the RESPONSE5 score for any whole blood sample. The derivation of the RESPONSE5 signature is described in the final section.

Construction of RNA-seq signatures

The two novel RNA-seq signatures presented in the manuscript, DISEASE and FAILURE, were both constructed using the Well-Fit Pairs (“WFP”) approach. This approach is an extension of the pairwise methodology used to derive the ACS COR that was fully described in [7]. The starting point for the WFP approach is a set of samples that can be broken into two groups (referred to generically as ‘cases’ and ‘controls’) and the corresponding gene-expression datasets. The algorithm then constructs a classifier to predict whether a given sample belongs to the case or control group. For the DISEASE and FAILURE signatures, the gene expression was measured in the form of RNA-seq exon-exon junction counts.

The first step in the algorithm is to perform a univariate feature selection analysis. Exon-exon junction counts were standardized using a panel of reference junctions, and the ability of each junction to differentiate cases from controls was assessed using the Wilcoxon test. A significance cutoff was applied and all junctions passing that cutoff were selected and carried forward to the next step, the pairwise selection step. The details of the univariate feature selection process differ slightly for the DISEASE and FAILURE signatures. For the DISEASE signature, the cases consisted of cures at baseline, and the controls were cures at week 24. These two groups were of approximately the same size, so univariate feature selection was straightforward. All cases and controls were compared, and all junctions with Wilcoxon p < 10^-20^ were selected. For the FAILURE signature, the cases were samples taken from treatment failures at pre-treatment baseline, and the controls were samples taken from cures at pre-treatment baseline. The controls outnumbered the cases in an 11:1 ratio. To prevent biased feature selection, 500 re-sampled training sets were created. Each training set consisted of the 7 treatment failures and 7 randomly selected cures. For each training set, the Wilcoxon p-value for each junction was computed. Junctions for which p < .01 in more than 70% (350/500) of the training sets were selected. For both the FAILURE and DISEASE signatures, the significance cutoffs were optimized using leave-one-out cross-validation (LOOCV).

After univariate feature selection, all possible pairwise combinations of selected features were formed. For each pair of selected junctions, a bivariate linear discriminant was constructed. Discriminants of the form $c_{1}*j_{1}+c_{2}*j_{2}+c_{3}$ were considered, where $j_{1}$ and $j_{2}$ are the standardized expression values for the two junctions and $c_{1},c_{2}$ and $c_{3}$ are unknown parameters fit via direct search. The sensitivity and specificity of the pairwise discriminant was computed for all combinations of $c_{i}\in\frac{a}{b}$ where $a$ and $b$ are in the set of integers from -10 to 10. The parameters $c_{2}$ and $c_{3}$were optimized by maximizing the sum of sensitivity and specificity. A cutoff was applied to sensitivity and specificity, and if the optimal bivariate discriminant had sensitivity and specificity both above the cutoff, the pairwise model was selected and included in the final ensemble. For both the DISEASE and the FAILURE signatures, the cutoff on sensitivity and specificity was 80%. The value for the cutoff was optimized using LOOCV.

After pairwise selection, the final signature consists of an ensemble of $N_{p}$ pairwise linear discriminant models. To assign a score to a given sample, the signature applies all $N_{p}$pairwise models to the sample. Each pairwise model cases a vote whether it thinks the sample looks like a case or a control. The overall score is then the percentage of the $N_{p}$votes that were for case. Samples with scores closer to 1 are more likely to be cases, samples with scores closer to 0 are more likely to be controls. All junctions (including the reference junctions used for standardization) for the DISEASE and FAILURE signatures are given in Tables S8 and S9, respectively. The full pairwise structure of the DISEASE and FAILURE signatures, including the parameters of the individual pairwise models, are given in Tables S11 and S12. For each pair of junctions in the table, the discriminant is computed as $d=c_{1}*j_{1}+c_{2}*j_{2}+c_{3}$, where $j_{1}$ and $j_{2}$ are the standardized expression values for the two junctions and $c_{1},c_{2}$ and $c_{3}$ are the coefficients listed in the table. If $d>0$, the pair votes ‘case’. The final score from the signature is then the percentage of pairs that voted ‘case’.

Adaptation of RNA-seq signatures to qRT-PCR

The DISEASE and FAILURE signatures were adapted to the qRT-PCR platform. Taqman assays were selected corresponding to all exon-exon junctions in the signatures, when possible. A table showing the assays selected for each junction is given in Table S13. Pairwise linear discriminant models were trained using the qRT-PCR Cts for all of the pairs in the DISEASE and FAILURE RNA-seq signatures in a direct search analogous to the method described above. Pairs including a junction without an available qRT-PCR assay were omitted. The pairwise structure of the qRT-PCR versions of the DISEASE and FAILURE signatures is given in Tables S14 and S15. The scores from the qRT-PCR strongly reproduced the RNA-seq signatures ability to discriminate baseline from week 24 among cures (for the DISEASE signature) and treatment failures from cures at baseline (for the FAILURE signature), as shown in Fig. S4.

Structure and implementation of RESPONSE5 signature

In order to construct an easily implementable transcriptomic signature, it is necessary to measure RNA abundance using a fast, affordable and targeted platform and to be measuring a minimal number of transcripts. The goal of the RESPONSE5 signature was therefore to create a qRT-PCR based signature that contains a small enough number of transcripts that it could conceivably be put onto a handheld device, and yet that simultaneously captures the strengths of the DISEASE and FAILURE signatures. The structure of the RESPONSE5 signature was tailored to meet these needs. In order to keep the number of transcripts in the signature low, it was desirable to remove the need for reference transcripts typically used to standardize the expression data. However, to be interpretable, raw Cts need some form of standardization to control for underlying variation in RNA abundance across samples. The RESPONSE5 signature thus standardizes data using ratios of signal transcripts that are being regulated in opposite directions, an approach we refer to as the Pair Ratio (PR) approach. In this case, the transcripts in the DISEASE signature are all higher in patients with more acute disease (i.e. decrease over the course of treatment and are higher in treatment failures than in cures, Fig. 1D), and the transcripts in the FAILURE signature are all higher in patients with less acute disease (i.e. increase over the course of treatment and are higher in cures than in treatment failures, Fig. 2D). So the individual entities that compose the RESPONSE5 signature are ratios of one transcript from the DISEASE signature to one transcript from the FAILURE signature. The specific assays used in the pairwise structure of the RESPONSE5 signature are given in Table S16.

The overall output of the RESPONSE5 signature on any whole blood sample is a score between 0 and 1. To compute this score, each of the six individual ratios in the signature first assign their own score to the sample, again ranging from 0 to 1. The final score is then the average over the scores from the individual pairs. A score close to 1 indicates that the sample appears to have acute disease and a score close to 0 indicates that a sample appears to be similar to healthy controls. One of the strengths of the signature is that it is robust to missing data, which often occurs in qRT-PCR measurement of expression. If a particular assay fails on a given sample, then the score is computed by simply computing the average score from all ratios that don’t involve that assay.

The only computational step in the signature is to convert each transcript expression ratio into a score between 0 and 1. The ratios are constructed such that samples with more acute disease have higher ratios. Thus, we just need a method that monotonically maps the ratio onto the interval 0 to 1, in effect deciding what values of the ratio should be considered ‘high’. This is done by comparing the ratio to the distribution of ratios present in the training set. The ratio is compared to all ratios from TB patients and controls in the CTRC. However, we don’t want the distribution of ratios to be dependent on the relative composition of our training set, which will not reflect the composition of the overall population. We therefore break the CTRC samples into five different groups: TB patients at baseline, TB patients at week 1, TB patients at week 4, TB patients at week 24, and healthy controls. To compute the score for a given ratio, we first compute the percentage of samples in each of the five CTRC groups that have scores lower than the given ratio. The score for that ratio is then the average over those five percentages. The result of this mapping procedure can be summarized in a look-up table. The score tables for the six pairs in the RESPONSE5 signature are given in Tables S17-S22. This methodology explicitly insures that the mapping of ratio to score does not depend on the relative number of samples in each of the five groups of our training set, so a repetition of the trial with a different composition of patients would lead to approximately the same mappings.

To summarize, below is the step-by-step procedure for computing the RESPONSE5 score for a whole blood sample:

1. Measure the Cts for the five assays listed in Table S16.
2. For each of the six pairs of assays in Table S16, compute the difference in raw Ct, which produces the log-transformed ratio of expression.
3. Compare the measured ratio to ratios in the look-up table for the given pair of transcripts in Tables S17-S22. Find the minimal ratio in column 1 of the table that is greater than or equal to the measured ratio.
4. Assign the corresponding score in the second column of the look-up table to the ratio. If the measured ratio is larger than all ratios in column 1 of the look-up table, then assign a score of 1 to the ratio.
5. The final score will be the average over the six scores thus computed for each ratio. If any assays failed on the sample, compute the average score over all ratios not including the failed assays.

Derivation of the RESPONSE5 signature

We have described the structure of the RESPONSE5 signature and how it assigns a score between 0 and 1 to any whole blood sample. In this section we describe how the size of the signature and particular pairs of assays were derived.

We began with the combined pool of qRT-PCR assays from the DISEASE and FAILURE signatures in Table S13. We formed all possible pairs of transcripts involving one transcript from DISEASE and one transcript from FAILURE, and for each pair of transcripts the log-transformed ratio of expression (difference in raw Ct) was computed for all samples in the CTRC.

Using this set of ratios, we sought to construct a minimal signature that optimally performs the two classifications on which the DISEASE and FAILURE signatures were trained: discriminating baseline from week 24 in micriobiological cures (DISEASE), and discriminating treatment failures from cures using baseline gene expression (FAILURE). To insure robustness across platforms, we considered the ability to make these two classifications using both RNA-seq and qRT-PCR data. Our optimization function was the mean over the four AUCs (two different platforms, two different classifications).

We started with the smallest possible signature involving only two transcripts. We found the pair of transcripts that maximized the optimization function (Table S5). Next, we carried out a greedy procedure of finding the individual transcripts that sequentially lead to the greatest increases in performance. Starting with the two transcripts in the best pair, we added all possible transcripts one at a time, formed all possible pairs involving the resulting three transcripts, and computed the optimization function. The transcript that led to the greatest increase in mean AUC was selected. This procedure was continued until the ensemble contained 8 assays (Table S5). No increase in optimization function was achieved by the addition of the sixth assay, so an ensemble size of five was selected. The transcripts that were added at each stage, the performance of the ensembles on both qRT-PCR and RNA-seq, and the values of the optimization function are all given in Table S5.
