# Supplementary figures and images for: Host blood RNA signatures predict the outcome of tuberculosis treatment
Source: Tuberculosis (Edinb). 2017 Dec;107:48–58. doi: 10.1016/j.tube.2017.08.004 (PMC5658513; doi:10.1016/j.tube.2017.08.004)

Sensitivity: Treatment Failures

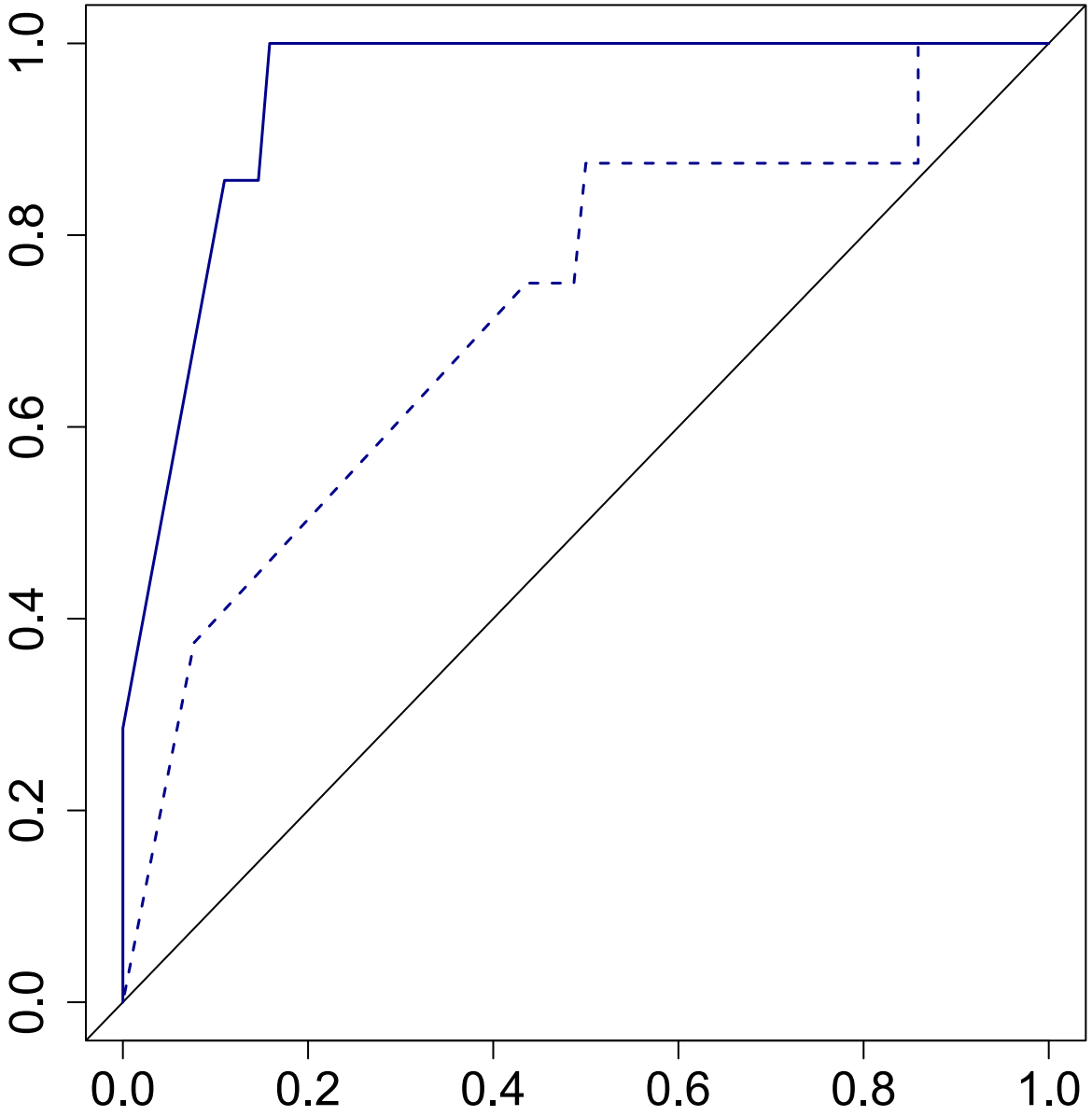

1 - Specificity: Cures

Supplement: Fig. S2 — RP11-295G20.2. [file mmc2.pdf]

**A.**

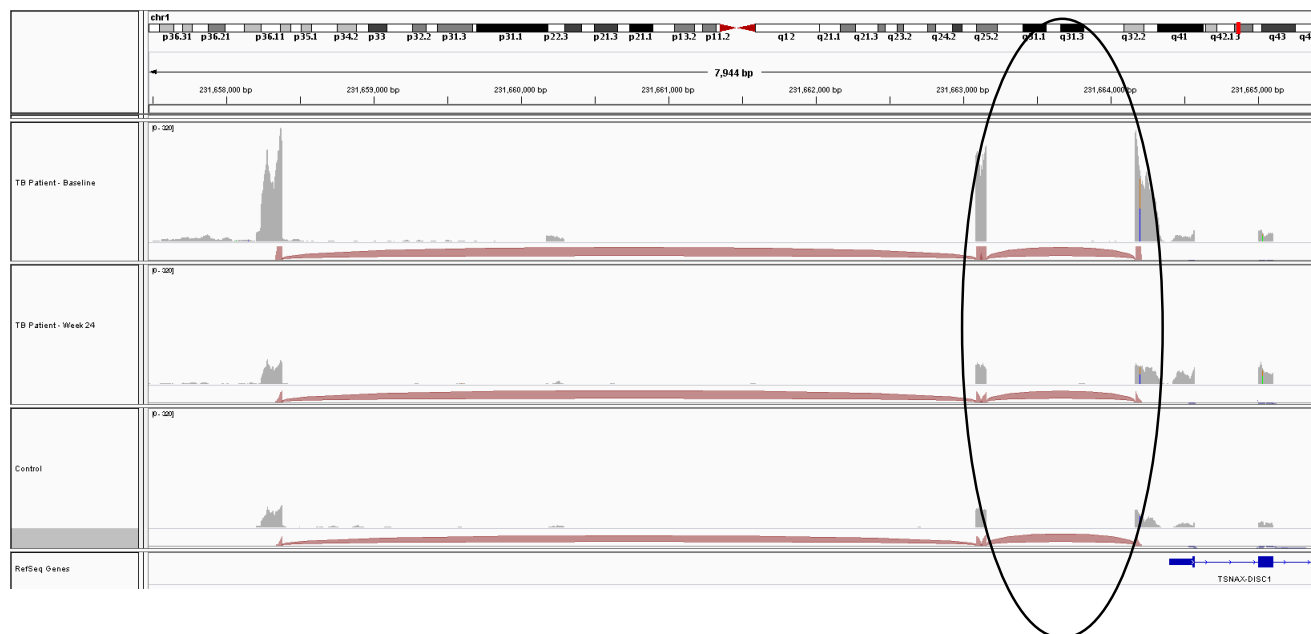

**B.**

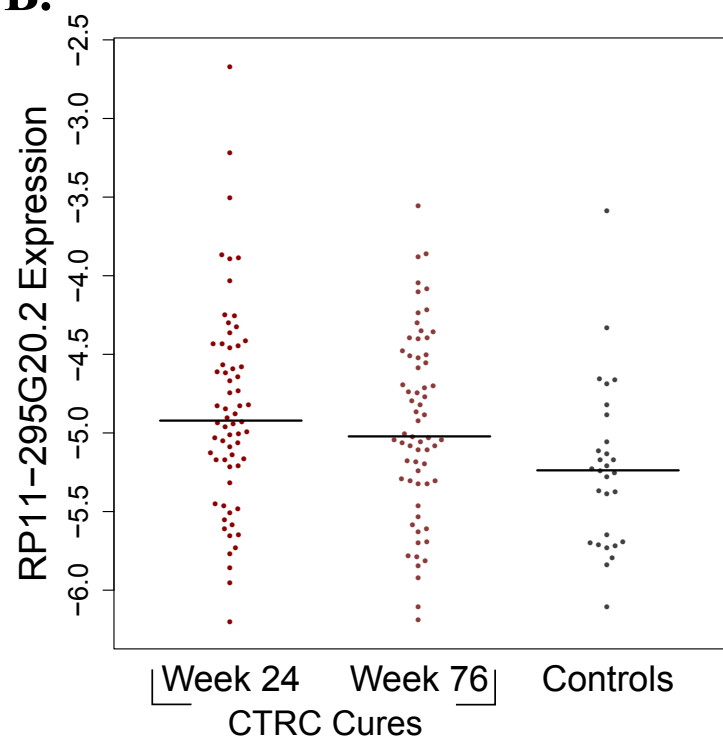

**C.**

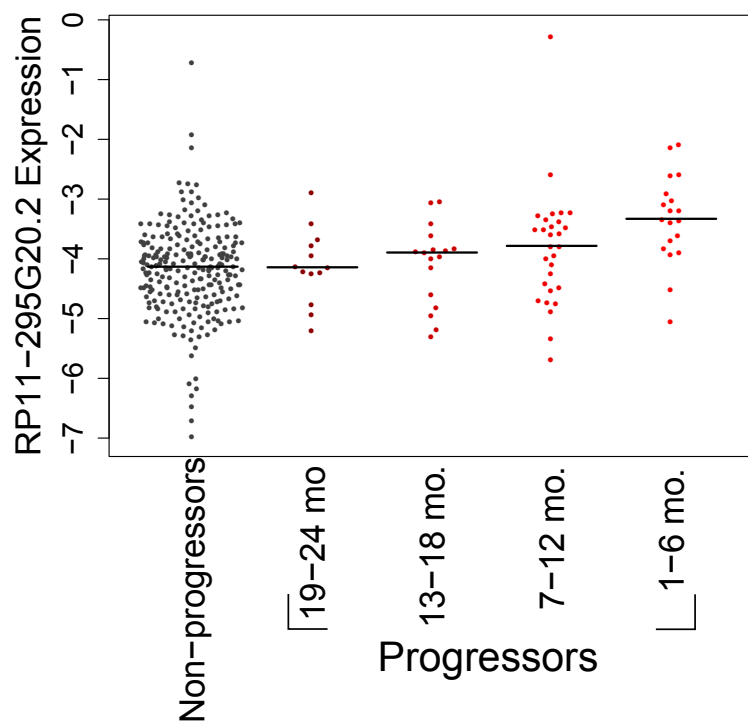

Supplement: Fig. S3 — DISEASE scores predict PET-CT resolution. [file mmc3.pdf]

Sensitivity: Non-resolved

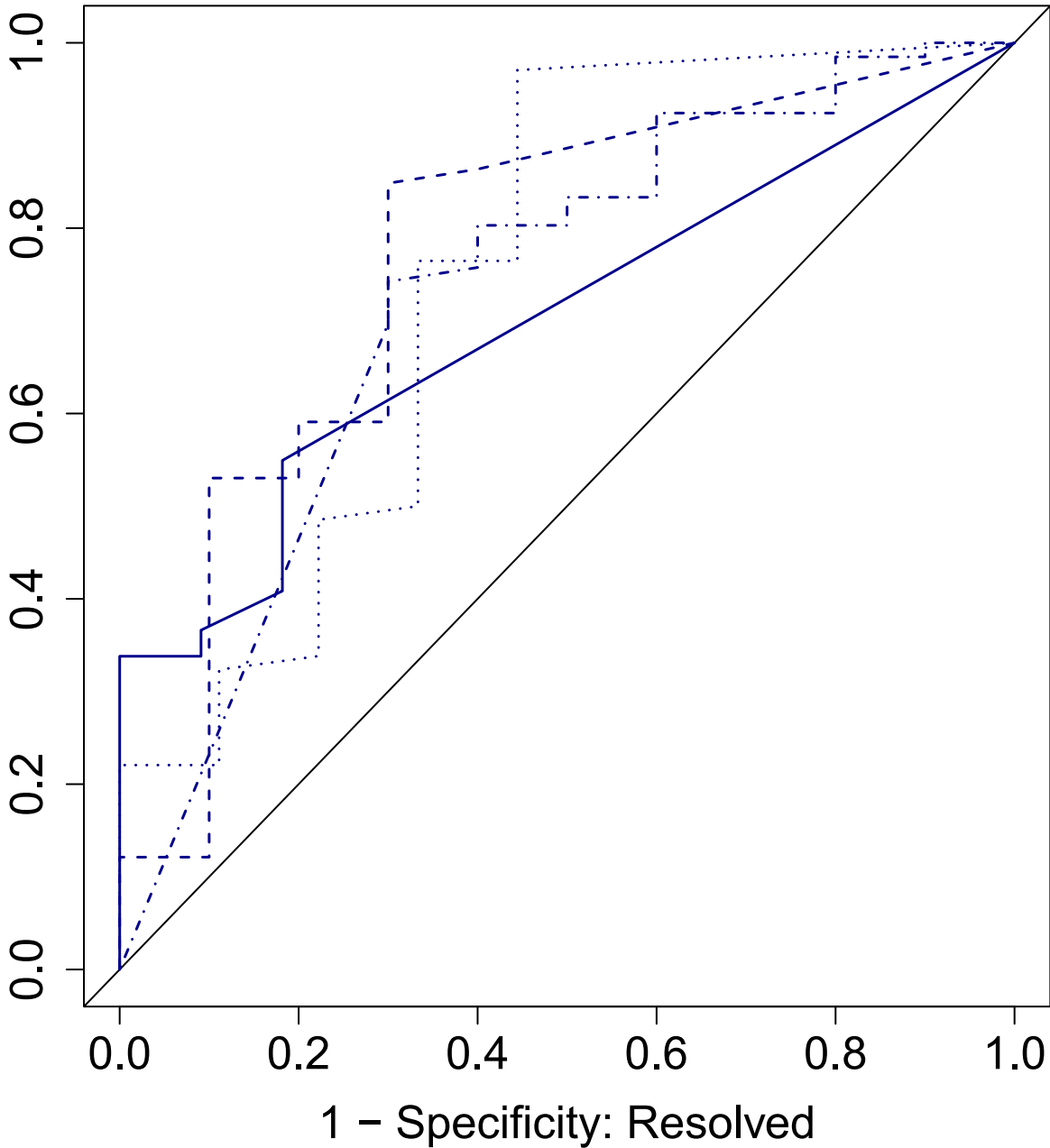

Supplement: Fig. S4 — DISEASE and FAILURE signatures were confirmed via qRT-PCR. [file mmc4.pdf]

**A.**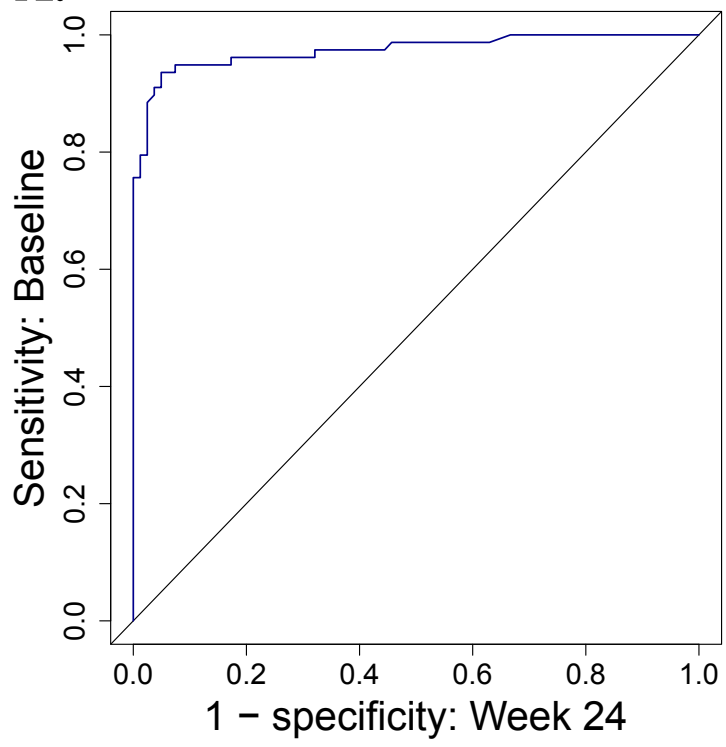**B.**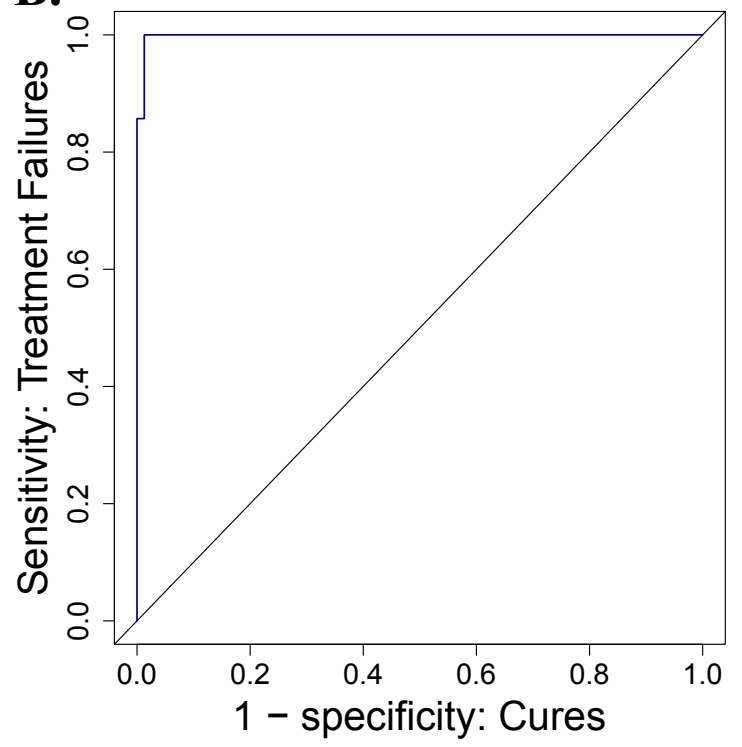

Supplement: Supplementary file 5 [file mmc5.pdf]
